# Some Rare Varieties

**Published:** 1920-05-15

**Authors:** 


### THE SEVERER FORMS OF LARYNGITIS.

### III. Some Rare Varieties.

(*b*) Chorditis Tuberosa: Singers' Nodes.

*Diagnosis.*—The voice shows impairment rather than actual hoarseness; the singer cannot strike the note truly, or hold it when actually obtained; again, his voice cracks on certain notes, or he is unable to pass smoothly from one register to another. He finds that his voice becomes tired much more easily than heretofore, and sometimes, after a hard day's work, it may fail altogether.

The node generally appears as a small, whitish, semi-translucent swelling, situated on the free edge of the cord, at the junction of the anterior and middle thirds; in most cases the condition is bilateral, the two nodes being placed *vis-à-vis*.

*Treatment.*—Treat any lesions of the higher air-passages that exist; absolute rest to the voice, with a simple open-air life for a few weeks or longer as the case may be. Sometimes it is wiser to remove the growth; this is a most difficult operation, and should, therefore, only be performed by a skilled laryngologist. Later on insist on proper voice-production, but prophylaxis would be much more valuable.

(*c*) Pachydermia.

*Diagnosis.*—Generally occurs in males who indulge in excessive smoking and drinking, or who strain their voices in dusty atmospheres, etc.

Two varieties are met with, viz.:

(1) Cup-and-ball variety. An oval swelling on the posterior extremity of the cord, in the immediate neighbourhood of the corresponding vocal process, this swelling being directed downwards and forwards from above and behind, so that its anterior end lies beneath the border of the cord.

(2) Inter-arytenoid variety. Here the approximation of the cords may be rendered impossible by the swelling, which also may so encroach upon the glottis that dyspnœa results. On phonation the free border of the growth assumes a crenated appearance; sometimes, also, a vertical fissure extends throughout the swelling and dried mucous crusts adhere to it.

In the case of (1) hoarseness is not marked, but with (2) it is very evident, and in this latter case dyspnœa also may be a prominent symptom.

Diagnosis of (1) is not difficult, seeing that such a cup-and-ball arrangement is not seen in the larynx in any other circumstances; but in the case of (2) the differentiation from tubercle is not always easy. However, the fact that in pachydermia the general health may be quite good, and also that the contour of the swelling is more regular, will help. The bilateral condition helps to differentiate this disease from early carcinoma, but if the inter-arytenoid region and vocal processes are involved, with impaired movements of the cords, the diagnosis may be most difficult.

*Treatment.*—Remove the cause if possible; absolute rest to the voice; apply cold baths to the skin over the larynx. Internally, potass. iodid. is sometimes useful, whilst alkaline and sulphurous waters are worth trying. Locally, dilute solutions of lactic acid, or iodine, or acid. salicylic, 5 to 15 per cent., dissolved in absolute alcohol, may prove efficacious. Remove the growth if deemed suitable for such treatment, or apply electrolysis. Inhalations of acetic acid, 2 to 3 per cent. solutions, are advised. Perhaps the most efficient treatment is the galvano-cautery when applied by a skilled and careful surgeon.

(*d*) Laryngitis Sicca: Chronic Atrophic Laryngitis.

*Diagnosis.*—Hoarseness, especially in the morning, when, after much coughing, the dried crusts of mucus are evacuated, and, concomitantly, the voice becomes clearer. No special pain is complained of; the breath may be very foul, due to the presence of inspissated crusts, and dyspnœa may occur from the same cause. The mucous membrane may or may not be anæmic, but the great characteristic is the diminution and inspissation of the natural mucus whose remains chiefly accumulate as dried crusts in the inter-arytenoid region.

*Treatment.*—Any lesions of the upper nasal tract should be treated; forbid excess of alcohol and tobacco; promote secretion by giving either potass. iodid. or ammonium carbonate or ammonium chloride; pilocarpine, grain, 1/20 to 1/10, three times a day, or apomorphine will promote a freer secretion of mucus and help to check the viscid accumulations. Locally, a warm laryngeal spray of a solution containing equal parts of sodium bicarbonate, sodium chloride, and borax will clear away many crusts, and if this be followed by a spray of menthol, 20 per cent. in paroleine, the patient will experience very great relief.

(*e.*) Chronic Sub-glottic Laryngitis.

*Diagnosis.*—Hoarseness occurs very early, gradually leading to complete aphonia; a barking, irritating cough is present, but the most characteristic symptom is gradually increasing dyspnœa. Again, patients are liable to subacute intercurrent attacks, which may so increase the swelling that urgent tracheotomy is called for.

Examination shows that the glottis is narrowed by a swelling below the cords, which narrows both the transverse and antero-posterior diameters; it is symmetrical on the two sides, and has a smooth surface. No glandular enlargements are found.

The diagnosis from rhinoscleroma can be made by the fact that in this latter disease the nose is generally affected as well, and also that the characteristic rhinoscleroma bacilli may be found; also that the swelling is much harder. Before making definite diagnosis, however, always try to exclude syphilis, tubercle, and carcinoma.

*Treatment.*—In early stages this should be on the same lines as for chronic catarrhal laryngitis, but potass. iodid. internally, in conjunction with inunctions of mercury, may also be very useful; give the former drug in small doses at first, carefully noting its effect on the swelling. The galvano-cautery and caustics have been used in a few cases, but the most satisfactory method of treatment appears to be tracheotomy, the patient wearing the tube for two months, or longer. Removal of the swelling, either by thyrotomy or by direct laryngoscopy, is feasible in some cases.

This disease is a rare one, and when present may well cause much anxiety to the practitioner.

8. Acute Crico-arytenoid Arthritis.

*Diagnosis.*—History of external trauma, or of a penetrating wound, or perhaps the swallowing of some hard

The Previous articles appeared February 14, p. 459 and March 27, p. 613.

**The Severer Forms of Laryngitis**—(*continued*).
and sharp substance; or of one of the specific fevers, especially influenza; or of a primary septic lesion in the neighbourhood which has now spread into the larynx; or of the unskilled use of laryngeal forceps. It may also result from syphilis, tubercle, lupus, or carcinoma.

The patient complains of pain in the throat and tenderness on the affected side; also hoarseness, dysphagia, and dyspnœa. In cases of bilateral lesions dyspnœa may occur suddenly, but the lesion is generally unilateral.

Examination shows that, in early cases, the mucous membrance—around and over the joint—is reddened; fixation of the cord on the same side will be seen, either in adduction or abduction.

At this stage the diagnosis is not difficult, the sudden onset following upon a possible cause, the swelling around the articulation, with a fixed position of the cord, being very characteristic.

*Treatment.*—Rest in bed, with complete rest to the voice; ice-bag or Leiter's coil locally, giving ice pellets to suck; apply leeches. Internally, potass. iodid., in increasing doses, is often very efficacious. Urgent dyspnœa will call for immediate tracheotomy; the later treatment cannot be discussed here.

